# An Intermediate Textbook of Physiological Chemistry, with Experiments

**Published:** 1918-02

**Authors:** 


**An Intermediate Textbook of Physiological Chemistry, With Experiments**, by C. J. V. Pettibone, Ph.D., Assistant Pro-..fessor of Physiological Chemistry, Medical School, University of Minnesota, Minneapolis. Cloth, 328 pages, price $2.50. St. Louis, C. V. Mosby Company, 1917.

The author intends that this textbook shall be supplemented by lectures, and used in this way it will serve the needs of advanced work as well as intermediate. It presents clearly and without tiresome details all the subjects usually included in the scope of biochemistry. Nearly half of the text is in the form of a laboratory manual covering most of the ground already treated in the didactic portion of the book. Reference to any subject is facilitiated by a separate index for each division of the book, supplemented by a very select bibliography. We notice that the latest developments of this branch of chemistry are mentioned, and the student is well oriented, providing he is properly grounded in the elementary principles of chemistry. The refinements of biochemical technic, as well as the discussion of doubtful theories and other mooted questions, are wisely excluded from this text.—F. W. B.

